# *WWP1* germline variants are associated with normocephalic autism spectrum disorder

**DOI:** 10.1038/s41419-020-2681-z

**Published:** 2020-07-23

**Authors:** Giuseppe Novelli, Antonio Novelli, Paola Borgiani, Dario Cocciadiferro, Michela Biancolella, Emanuele Agolini, Marco Pietrosanto, Rosario Casalone, Manuela Helmer-Citterich, Emiliano Giardina, Suresh K. Jain, Wenyi Wei, Charis Eng, Pier Paolo Pandolfi

**Affiliations:** 1 Department of Biomedicine and Prevention, Tor Vergata University of Rome, 00133 Rome, Italy; 2 IRCCS Neuromed, Pozzilli (IS), Italy; 3 Department of Pharmacology, School of Medicine, University of Nevada, Reno, NV 89557 USA; 4 Laboratory of Medical Genetics, Bambino Gesù Children’s Hospital, IRCCS, Rome, Italy; 5 Department of Biology, Tor Vergata University of Rome, 00133 Rome, Italy; 6 Cytogenetics and Medical Genetics Laboratory, Ospedale di Circolo, ASST Sette Laghi, Varese, Italy; 7 Molecular Genetics Laboratory UILDM, Santa Lucia Foundation, 00142 Rome, Italy; 8 Intonation Research Laboratories Pvt. Ltd, Hyderabad, Telangana 500076 India; 9 Cancer Research Institute, Beth Israel Deaconess Cancer Center, Harvard Medical School, Boston, MA 02215 USA; 10 Department of Pathology, Beth Israel Deaconess Medical Center, Harvard Medical School, Boston, MA 02215 USA; 11 Genomic Medicine Institute, Lerner Research Institute, Cleveland Clinic, Cleveland, OH 44195 USA; 12 Taussig Cancer Institute, Cleveland Clinic, Cleveland, OH 44195 USA; 13 Department of Genetics and Genome Sciences, Case Western Reserve University School of Medicine, Cleveland, OH 44106 USA; 14 Germline High Risk Cancer Focus Group, Case Comprehensive Cancer Center, Case Western Reserve University, Cleveland, OH 44106 USA; 15 MBC, Department of Molecular Biotechnology and Health Sciences, University of Turin, Turin, TO 10126 Italy; 16 DRI, Renown Health, Nevada System of Higher Education, Reno, NV 89512 USA

**Keywords:** Cognitive neuroscience, Genetics of the nervous system, Neuroscience

Dear Editor,

Autism spectrum disorder (ASD, MIM: 209850) is a group of common but heterogeneous neurodevelopmental disorders with a prevalence of 4–10 per 10,000 individuals^1,2^. About 5% of ASD cases are caused by single-gene variants in *FMR1* (MIM: 309550)*, MECP2* (MIM: 300005), or *SHANK3* (MIM: 606230); 10% by copy number variants (CNVs)^2^, while the majority is attributed to polygenic inheritance of common variants^3^. In addition, germline *PTEN* mutations have been identified in 2–5% of all ASD patients and ~10% of macrocephalic ASD^4^. Recently, Lee et al.^5^ identified germline variants within the E3 ubiquitin ligase *WWP1* (MIM: 602307) gene in *PTEN* mutation negative individuals with neoplastic phenotypes found in PHTS (MIM: 158350).

To establish whether WWP1 could play a role in ASD and neurodevelopment disorders, we analyzed 198 unrelated individuals mainly referred for syndromic or non-syndromic developmental delay and/or ASD of unknown genetic etiology. All individuals were clinically diagnosed with ASD on the basis of the fourth edition of the Diagnostic and Statistical Manual of Mental Disorders (DSM-IV). Whole-exome sequencing, validated by Sanger sequencing, identified eight different heterozygous germline mutations (one recurrent in three unrelated patients) of the *WWP1* gene in 10 of 198 unrelated probands via WES (Table 1). None of the variant positive probands had macrocephaly. In two cases, parental origin could not be investigated, therefore, a de novo origin of the mutation, cannot be ruled out. For each patient (6 males and 4 females; ages 3–26), the clinical data have been reassessed. None of the probands had germline *PTEN* mutations or other mutations in genes (*FMR1, SHANK3, MECP2, CDK19*) associated with ASD/intellectual disability (ID). We independently confirmed that *WWP1* variation does not act as a modifier for ASD phenotypes in PHTS with none of ~600 mainly American *PTEN* mutation positive research associated with the *WWP1* locus. Similarly, routine chromosome studies and *FRAXA* locus were normal. GnomAD database analysis revealed that the identified WWP1 variants with the exception of R389S, R893H, and M728L (never detected), existed with a cumulative frequency of 0.00085 in ethnically matched populations (EUR), indicating that they are very rare variants. Specifically, *WWP1* germline variants occurred in 10/396 alleles (allelic freq. = 0.0252) from the 198 unrelated individuals with ASD/ID (Table 1) which is a highly significant difference from European population frequencies from GnomAD (*p* < 0.00001; OR = 30.6 with 95% CI 16.27 and 57.59). We therefore extended the study to a cohort of 1158 individuals from the Italian general population to establish the frequency of *WWP1* variants in this Italian cohort. We detected three *WWP1* rare variants (c.1118G>A, p-Arg373Gln; c.1486G>C, p.Glu496Gln; c.2234A>G, p.Asn745S) (3/2316 alleles: allelic freq. = 0.00129). Notably, *WWP1* variants were again shown to be over-represented in the ASD/ID series, even when compared with the Italian cohort examined (*p* < 0.00001; OR = 19.93 with 95% CI 5.47 and 72.90). The variants are found in all functional domains of the protein (the catalytic C-terminal HECT domain; the N-terminal C2 domain and WW domains) with an over-representation in the HECT domain (4/8). To predict the potential impact of the identified variants on the protein we used different tools (PolyPhen2, Mutation Taster, SIFT, MetaLR_pred, and MetaSVM_pred). The recurrent N745S variant has been previously reported by Lee et al.^5^: it is in the HECT domain and is expected to decrease its binding to the N-terminal domain. Analogously, R86H (C2 domain) was also described by Lee et al.^5^. This variant is functionally relevant since it induces a gain-of-function effect in triggering PTEN polyubiquitination^5^. With regards to the other five coding variants observed in our ASD cases, one is predicted by in silico analysis to be deleterious (R528H), while the others gave conflicting results.

**Table 1:** Summary of variations in ASD patients carrying *WWP1* mutations.

Patient ID	Sex	Exon	Position (Hg19)	Nucleotide	Amino acid	Domain	GnomAd^a^	dbSNP	Transmission
GM4277	F	Int 7	87414243	c.540-5T>C		NA	0.0027	rs187132881	Mother
GM3474	M	11	87439881	c.1167A>C	p.Arg389Ser	WW1	0	NA	NA
A020	M	14	87443954	c.1583G>A	p.Arg528His	WW4	0.000008	rs554041348	Father
GM6802	F	20	87460703	c.2234A>G	p.Asn745Ser	HECT	0.00003	rs148651938	Mother
GM8105	M	20	87460703	c.2234A>G	p.Asn745Ser	HECT	0.00003	rs148651938	Father
GM-1HSL	M	20	87460703	c.2234A>G	p.Asn745Ser	HECT	0.00003	rs148651938	NA
GM4098	F	20	87460645	c.2176G>A	p.Val726Ile	HECT	0.000023	rs144129917	Mother
GM8302	F	25	87479031	c.2678G>A	p.Arg893His	HECT	0	rs755897749	Father
A036	M	20	87460651	c.2182A>T	p.Met728Leu	HECT	0	NA	Father
A069	M	5	87393781	c.257G>A	p.Arg86His	C2	0.000023	rs371650373	Mother

^a^EUR.

Our results suggest that germline *WWP1* variants identified in ASD/ID/NDDs may contribute to the pathogenesis of ASD/ID/NDDs. In addition, since the enzymatic activity of WWP1 can be inhibited by the natural compound, indole-3-carbinol^6^, our study identifies a possible therapeutic target for individuals with ASD/ID/NDDS.

## Web resources

GnomAD, https://gnomad.broadinstitute.org/

PolyPhen2, http://genetics.bwh.harvard.edu/pph2/

Mutation Taster, http://www.mutationtaster.org/

SIFT, https://sift.bii.a-star.edu.sg/

OMIM, https://OMIM.org/.
